# A rare case of solitary fibrous tumor of the lung parenchyma: case report

**DOI:** 10.1093/jscr/rjae426

**Published:** 2024-06-24

**Authors:** Sumayya Afreen, Xiyi Wu, Maria Emilia Espinoza Miranda, Milenko Lazarevic

**Affiliations:** Department of Internal Medicine, Deccan College of Medical Sciences, DMRL X Road, 500058 Hyderabad, India; Department of Internal Medicine, The Chinese University of Hong Kong, Shatin 00000, New Territories, Hong Kong; Department of Internal Medicine, University of Cuenca, Cuenca 01010, Ecuador; Department of Internal Medicine, Swedish Hospital, 5140 N. California Ave, Chicago, IL 60625, United States

**Keywords:** solitary fibrous tumor, lung mass, lung parenchyma, case report

## Abstract

Solitary fibrous tumor (SFT) of the lung is a rare neoplasm, usually originating from lung pleura. We present a case report of a 57-year-old male with no significant medical history who was incidentally diagnosed with an SFT of lung parenchyma on chest computed tomography scan. Radiological imaging revealed a well-defined mass in the left lower lobe of the lung. Biopsy and histopathological examination confirmed the diagnosis of solitary fibrous tumor. This case highlights the importance of considering SFT in the differential diagnosis of lung masses, as its clinical presentation and radiological features can mimic those of more common pulmonary malignancies.

## Introduction

Solitary fibrous tumors (SFT) are a rare subset of mesenchymal neoplasms, typically incidental findings on imaging studies that can arise from various anatomical locations, including the pleura, soft tissues, and, albeit less commonly, the lung parenchyma [1]. The incidence of SFT is so rare that only >800 cases have been reported worldwide [2]. Despite their historical association primarily with the pleura [3], a few cases originating from lung parenchyma have been reported recently [4]. Here we report a rare case of SFT of the lung parenchyma.

## Case presentation

**Figure 1:**
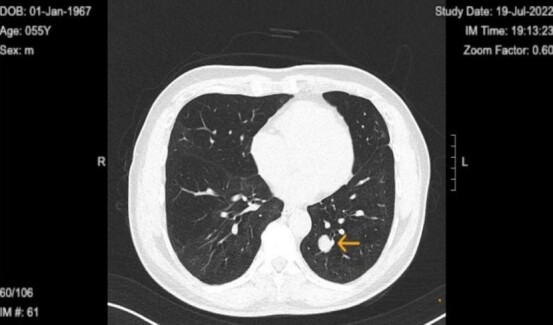
Chest computed tomography in 2022 revealed well circumscribed solitary lung tumor of 1.5 × 1.3 cm in size.

The patient is a 57-year-old male in the United States, who is a non-smoker with good past health. He had no history of occupational exposure to silica, beryllium, or asbestos. He presented with fever and dyspnea and attended the ER, where he was diagnosed with COVID-19 infection and a lung nodule. He underwent a computed tomography (CT) scan of the chest as a part of their diagnostic workup for suspected COVID-19 infection. Chest CT on 21 May 2021 revealed the presence of a well-demarcated non-calcified solid nodule of 1.5 × 1.3 cm in the left lower lobe of the lung (Fig. 1). It was suspected to be a lung carcinoma at first. CT-guided lung biopsy was recommended for further diagnosis.

A successful CT-guided left lower lobe lung nodule biopsy was performed on 17 February 2022. The patient was positioned prone on the CT scanner for a limited scan to locate a lung mass. A 19-gauge coaxial needle was used under sterile technique, guided by CT, to obtain three 20-gauge core biopsies from a 1.5 cm nodule in the left lower lobe. Adequate material was collected for cytologic evaluation, and a post-biopsy CT showed no pneumothorax. The procedure was well tolerated.

The histopathology findings revealed a spindle cell proliferation with a bland appearance [5], accompanied by surrounding hyalinized stroma. Immunohistochemistry demonstrated positivity for STAT 6 in the spindle cells, consistent with a diagnosis of SFT. Conversely, staining for pan-cytokeratin, S100, and smooth muscle actin yielded negative results. Cytology was benign, with no mitotic activity or tumor necrosis seen.

**Figure 2:**
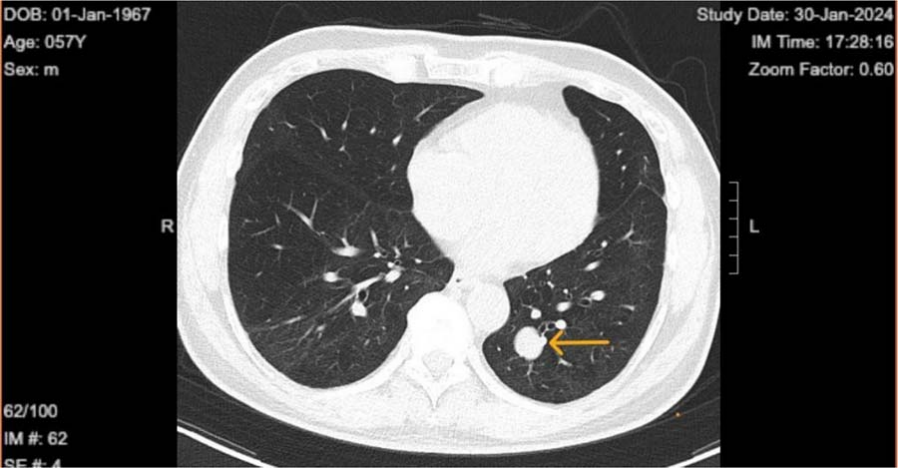
Chest computed tomography in 2024 shows well circumscribed solitary lung tumor of 1.9 × 1.6 cm in size.

**Figure 3:**
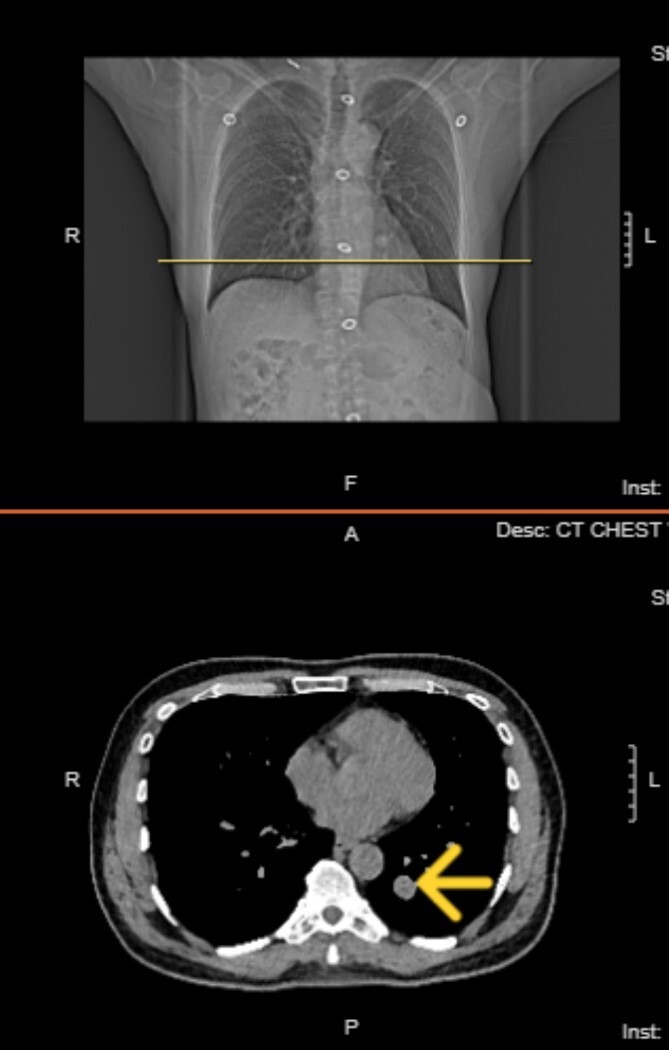
Top image: Coronal view indicating the level of the solitary fibrous tumor (SFT). Bottom image: Axial CT scan at the indicated level, showing a well-circumscribed, homogeneous mass.

Upon diagnosis of SFT, the patient was referred to a surgeon for consideration for left lower wedge resection via video-assisted thoracoscopic surgery. However, the treatment consisted of monitoring of the tumor with biannual CT scans. Immediate surgical intervention was not performed, as the tumor remained stable in size, asymptomatic, and showed no signs of aggressive behavior. The latest CT on 30 January 2024 shows the tumor size to be 1.9 × 1.6 cm (initial size 1.5 × 1.3 on 21 May 2021) with no accompanying symptoms (Figs 2 and 3).

## Discussion

SFT is a mesenchymal neoplasm that typically arises from the visceral pleura, accounting for 60% to 80% of reported cases [3, 6]. However, its occurrence in the lung parenchyma is exceedingly rare. Our case adds to the limited literature on SFT originating within the lung parenchyma.

Traditionally considered a tumor of mesothelial origin, SFT was initially described in the pleura by Klemperer and Rabin in 1931 [7]. Since then, the pleura have remained the most common site of SFT occurrence. Clinical features mostly depend on the site and size and malignant potential of the tumor [8]. SFT of the pleura occurs mainly in adults. The sex incidence is equal and they are seen in all age groups, most commonly presenting in the 60s and 70s. The etiology is unknown, and smoking and asbestos exposure are not correlated with SFT pathogenesis.

In our case, the identification of SFT within the lung parenchyma underscores the importance of considering this entity in the differential diagnosis of pulmonary masses, particularly in cases with atypical radiological or histological features. Radiologically, SFT of the lung may manifest as a well-defined, heterogeneous mass with enhancement, mimicking other primary lung tumors or metastatic lesions. Histopathological examination remains the mainstay for definitive diagnosis, with characteristic features including spindle-shaped cells arranged in a pattern-less architecture and positive immunostaining for CD34, Bcl-2, and CD99 [9].

The pathogenesis of SFT in the lung parenchyma remains poorly understood, with hypotheses suggesting a possible mesenchymal origin or intraparenchymal extension from adjacent pleural-based lesions. Recent molecular studies have identified recurrent NAB2-STAT6 gene fusions in both pleural and parenchymal SFT [10], suggesting a common genetic basis for tumor development. However, further research is needed to elucidate the precise mechanisms driving SFT tumorigenesis and identify potential therapeutic targets. About 20% of SFTs are reportedly malignant [11].

The optimal management of lung parenchymal SFT relies on a multidisciplinary approach, incorporating radiological imaging, histopathological examination, and surgical intervention. Surgical resection remains the main treatment for localized disease, but long-term surveillance is crucial due to the risk of recurrence and metastasis [12].

## Conclusion

In conclusion, SFT is a rare mesenchymal neoplasm that predominantly arises from the pleura but can infrequently occur within the lung parenchyma. Recognition of this entity in the lung parenchyma necessitates awareness of its distinct clinical and pathological features and careful consideration in the differential diagnosis of pulmonary masses. Continued reporting and investigation of lung parenchymal SFT are essential to enhance our understanding of its pathogenesis and improve patient outcomes.
